# Abscisic acid-regulated microRNA biogenesis through HYPONASTIC LEAVES1

**DOI:** 10.1093/plcell/koad135

**Published:** 2023-05-16

**Authors:** Peng Liu

**Affiliations:** Assistant Features Editor, The Plant Cell, American Society of Plant Biologists, USA; Donald Danforth Plant Science Center, Saint Louis, MO, USA 63146

The central dogma of molecular biology describes the genetic information flow from DNA to RNA to protein (Crick 1958). Since then, many noncoding RNAs and other exceptions to the dogma have been discovered and found to play critical roles in diverse biological processes without expressing proteins (Cech and Steitz 2014). In new research conducted by **Junghoon Park and colleagues** (Park et al. 2023), the authors studied the biogenesis of microRNAs (miRNAs), a type of noncoding RNAs that post-transcriptionally regulates gene expression. They found that HYPONASTIC LEAVES1 (HYL1), a core protein of the miRNA biogenesis machinery, is essential for regulating miRNA expression in response to abscisic acid (ABA) signaling in Arabidopsis.

Using a luciferase reporter system to monitor miRNA production, the authors performed a forward genetic screen to isolate Arabidopsis mutants with impaired miRNA biogenesis and activity. One of the mutants is mapped to *HIGH EXPRESSION OF OSMOTICALLY RESPONSIVE 15* (*HOS15*). A *hos15* mutant was previously identified in a screen for mutations with altered expression of stress-induced genes (Zhu et al. 2008). The new *hos15* mutant allele isolated by Park et al. displayed several morphological phenotypes, such as delayed flowering and shortened stems. Complementation confirmed that *hos15* was the causal mutation for these phenotypes. To investigate the role of HOS15 in the miRNA pathway, the authors tested protein-protein interactions between HOS15 and several miRNA biogenesis factors. Only HYL1 showed an interaction with HOS15, indicating HOS15 regulates miRNA processing through HYL1.

*HOS15* encodes a nuclear protein with dual functions as an E3-ligase substrate receptor and a transcriptional co-repressor protein. The authors suggest that the E3-ligase substrate receptor function of HOS15 is not involved in the miRNA pathway because HYL1 degradation is independent of E3-mediated ubiquitination. Therefore, they hypothesized that HOS15 works as a transcriptional co-repressor in the miRNA pathway. They began to test this hypothesis by assessing the interaction between HYL1 and known partners of HOS15, using yeast-2-hybrid and co-immunoprecipitation assays. They discovered that HYL1 interacts with 2 histone deacetylases: HDA9 and HDA15. HOS15-HDA9 is a well-defined stress-responsive complex (Park et al. 2018). Furthermore, HYL1 influences epigenetic marks, as evidenced by the increased levels of histone acetylation observed in *hyl1*, *hda9*, and *hos15* mutants.

Chromatin immunoprecipitation sequencing (ChIP-Seq) analysis revealed that both HOS15 and HDA9 bind to many *MIRNA* loci. The bound loci did not have any obvious sequence motifs in common. The authors also conducted ChIP-qPCR assays to test the recruitment of HOS15 to *MIRNA* loci and observed that such associations were lost in the *hyl1* mutant, suggesting HYL1 plays a crucial role in this process. Interestingly, ABA treatment facilitates this recruitment, which ultimately triggers histone deacetylation. The authors also tested the interactions of HYL1 with *MIRNA* loci after eliminating the nascent miRNA primary transcripts (pri-miRNA) from the loci. Results showed that the association of HYL1 with *MIRNA* loci is dependent on the nascent pri-miRNAs. In summary, HYL1 serves as a scaffold to direct the HOS15-HDA9 complex to *MIRNA* loci (Figure).

**Figure.**
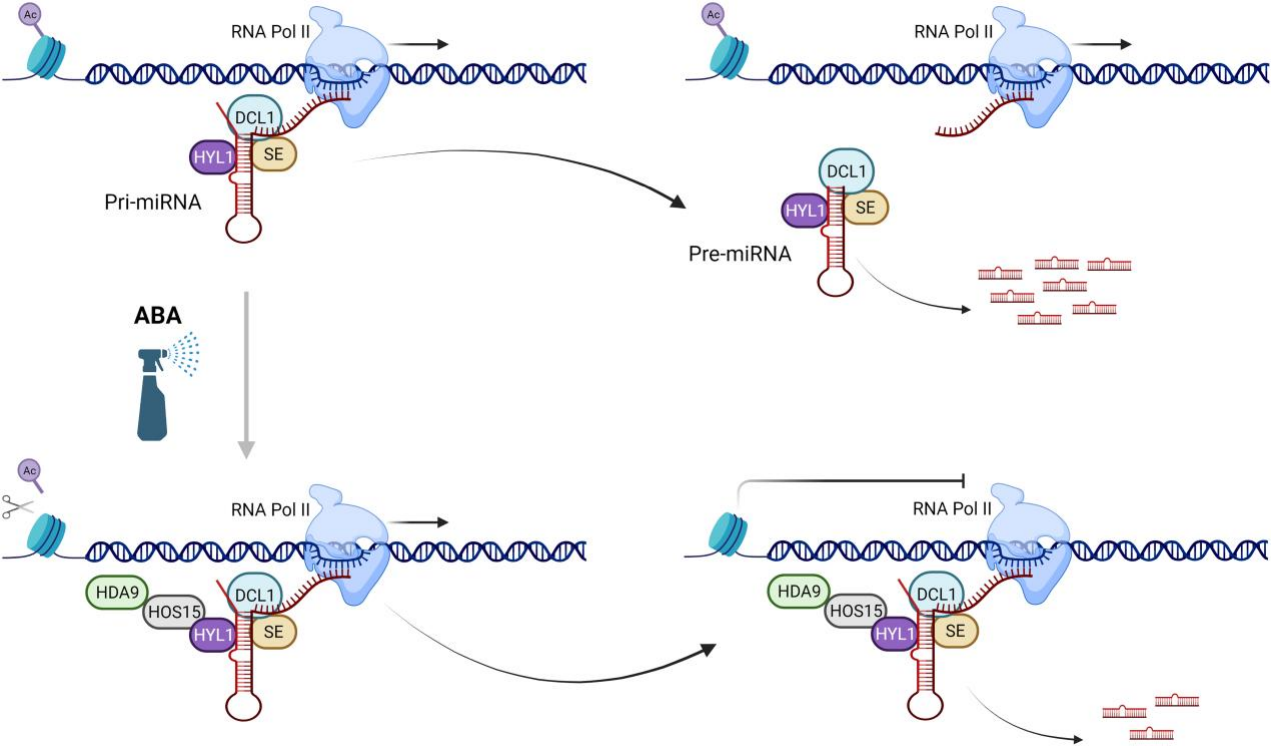
HYL1 recruits the HOS15-HDA9 complex to *MIRNA* loci to regulate miRNA processing. *MIRNA* loci can be transcribed by Pol II to produce a pri-miRNA that forms a hairpin structure. The processing complex includes HYL1 and SERRATE (SE) that bind with pri-miRNA and trigger the production of mature miRNAs. Under ABA treatment, HYL1 acts as a scaffold to recruit HOS15 and HDA9 to *MIRNA* loci, which in turn changes the histone acetylation profile and represses miRNA production. Created with BioRender.com.

The authors observed a minor effect on miRNA levels in *hos15* and *hda9* mutants grown under control conditions. However, when treated with ABA, *hos15* and *hda9* mutants displayed a moderate increase in the abundance of many miRNAs. Surprisingly, no noticeable change was found in pri-miRNA levels in either mutant. To investigate this inconsistency between pri-miRNA level and mature miRNA accumulation, a RNA polymerase II (Pol II) occupancy assay was performed. Significant enrichment of Pol II at *MIRNA* loci in both control and ABA conditions was observed in *hos15* and *hda9* mutants, indicating an enhancement of pri-miRNA transcription.

This study proposes an intriguing model (Fig.) by which plants regulate ABA-mediated miRNA production. The HOS15-HDA9 complex uses HYL1 and pri-miRNA as the scaffold to target the upstream of *MIRNA* loci, remove histone acetylation marks, and repress the miRNA processing. Considering the known functions of HYL1 as a regulator of transcription and stability, this work shows that HOS15-HDA9 regulates some *MIRNA* genes at both transcriptional and processing levels upon interaction with HYL1.
